# Psychological Safety and Work Design as Mediators of Supervisors’ Dark Triad Traits Impact on Nurses’ Task Performance

**DOI:** 10.3389/ijph.2024.1607340

**Published:** 2024-11-01

**Authors:** Andrés Raineri, Macarena Cartes

**Affiliations:** ^1^ School of Business Administration, Pontificia Universidad Católica de Chile, Santiago, Chile; ^2^ Master of Health Administration (MHA), Pontificia Universidad Católica de Chile, Santiago, Chile

**Keywords:** psychological safety, dark triad traits, task performance, enriched work design, nurses work environment

## Abstract

**Objectives:**

This study investigates how nurse supervisors’ Dark Triad personality traits (Machiavellianism, narcissism, psychopathy) influence nurses’ task performance, mediated by perceptions of enriched work design (autonomy, task variety, social support, safe work conditions, feedback quality) and psychological safety.

**Methods:**

A multisource approach was used to collect data from 256 manager-nurse dyads across various healthcare settings. Nurses completed surveys assessing their work design and psychological safety. Managers completed a self-assessment of Dark Triad traits and rated their nurse subordinates’ task performance. Confirmatory factor analysis and structural equation modeling (SEM) were used for analysis.

**Results:**

Supervisors’ Dark Triad traits core component impacted nurses’ task performance indirectly, mediated by psychological safety and nurses’ perceptions of their enriched work design. Psychopathic traits revealed a significant direct negative effect on nurses’ performance, while other Dark Triad traits did not show direct effects.

**Conclusion:**

This study sheds light on key factors influencing nurses’ performance, offering insights for healthcare organizations aiming to optimize work environments and improve team effectiveness.

## Introduction

In healthcare organizations, the performance and wellbeing of nursing professionals are critical for the effective delivery of patient care. As a result, various lines of research have focused on identifying the factors that influence nurses’ performance and wellbeing [1–3]. Key determinants include work design, psychological safety, individual and interpersonal wellbeing, nurse staffing, availability of patient care resources, and the personality traits, management styles, and behaviors of supervisors. In the healthcare literature, such comprehensive environments are frequently described as “better care work environments” [1], “healthy work environments” [2], or “positive work environments” [4]. Collectively, these factors are often referred to in the literature as the nurses’ work environment. These broader conditions have been identified as key determinants of nurses’ wellbeing and performance [1, 3, 4].

One component of nurses’ work environment is their work design. Specific dimensions of work design—such as decision-making autonomy, social support, performance feedback, safety work conditions, and skill variety—have been shown to significantly enhance nurses’ wellbeing and performance [5–9]. Work designs featuring high levels of these attributes are often described as having an “enriched work design” [10–12]. Such enriched work designs not only support nurses’ professional needs but also contribute to their overall job satisfaction and performance.

Another component of nurses’ work environment are the leadership styles, behaviors, and personality traits of nurses’ leaders or managers. Such attributes play a pivotal role in either reinforcing or undermining nurses overall work environments [2–4]. Leadership styles such as transformational leadership, inclusive leadership, and abusive supervision have been recognized as significant predictors of nurses’ wellbeing and performance [13–17]. Recent studies have increasingly highlighted the role of leaders’ nefarious personality traits, specifically those within the Dark Triad—Machiavellianism, narcissism, and psychopathy—in shaping workplace dynamics and outcomes. These traits, characterized by manipulation, self-centeredness, and a lack of empathy, have been linked to negative employee outcomes, such as decreased job satisfaction, increased stress, and impaired psychological safety, in various organizational contexts [18, 19]. Leaders exhibiting Dark Triad traits may foster a toxic work environment marked by fear, hostility, and mistrust [20, 21], significantly undermining the wellbeing and performance of their subordinates.

Finally, psychosocial factors, such as psychological safety and burnout, have been considered part of nurses’ work environment [22–24], and shown to be affected by nurses’ work design and their supervisors’ styles and traits. In turn these psychosocial factors have been identified as antecedents of nurses’ wellbeing and performance [22–25].

Despite the growing body of research on the effects of work design, psychosocial factors, and leadership characteristics on employees’ performance and wellbeing, limited attention has been given to the impact of managers’ Dark Triad traits within specific industries, including healthcare. Initial findings suggest that these traits may have differential impacts across different industries [26]. This gap is significant, considering the direct link between nursing professionals’ performance and the quality and safety of patient care. Understanding how these personality traits influence nurses’ experiences could provide valuable insights into preventing negative workplace environments and enhancing overall healthcare delivery.

To address this gap, the current study aims to explore the mechanisms through which managers’ Dark Triad traits affect nurses’ psychological safety, perceptions of their work design, and task performance. By examining these relationships, this research seeks to offer a more comprehensive understanding of how managers Dark Triad traits influence nurses’ wellbeing and performance, ultimately contributing to more effective management practices in healthcare settings.

### The Importance of Nurses’ Work Environment in Their WellBeing and Performance

In healthcare organizations, the wellbeing and performance of nursing professionals are essential components that directly influence the quality of patient care and outcomes. Nurses are often at the frontline of patient interactions, providing critical care, monitoring patient health, and executing medical interventions. As such, their physical, emotional, and psychological wellbeing significantly impacts patient safety, satisfaction, and recovery rates. Studies have shown that when nurses experience high levels of job satisfaction and wellbeing, there is a marked reduction in medical errors, improved patient outcomes, and increased overall patient satisfaction [27, 28]. Conversely, poor wellbeing among nurses can lead to burnout, high turnover rates, and suboptimal patient care, which pose substantial risks to healthcare quality [27, 28].

Ensuring a supportive work environment for nurses is not just beneficial for the staff but also crucial for maintaining high standards of patient care. The nurses’ work environment is a comprehensive concept that encompasses a wide range of factors influencing nurses’ experiences and interactions within the workplace [1–4]. These factors can be structural, such as work design, nurse staffing levels, and the availability of patient care resources, or interpersonal, including the quality of relationships with colleagues, communication, and the management styles and personality traits of supervisors. Other elements like organizational culture, leadership support, professional development opportunities, and work-life balance also play a critical role in shaping the environment in which nurses operate. Together, these determinants influence how nurses perceive their work environment, impacting their engagement, satisfaction, wellbeing, and ultimately, their performance [1–4].

### Enriched Work Design in Nursing

One component of nurses’ work environment is the design of their work. Enriched work design refers to job characteristics that provide greater autonomy in decision-making, a variety of skills, social support, safe working conditions, and meaningful feedback, among other features [5, 12]. In the context of nursing, enriched work design has been associated with higher levels of job satisfaction, wellbeing, and performance [7, 8]. Attributes such as autonomy allow nurses to make decisions and execute tasks independently, promoting a sense of control over their work and professional accomplishment [2, 3, 29]. Nurses’ skill variety and opportunities for continuous professional development keep their job stimulating, preventing burnout and fostering a sense of competence [9, 23]. Social support from colleagues and supervisors provides emotional and professional assistance, facilitates collaboration and teamwork, reduces feelings of isolation and enhances job satisfaction, work performance, and quality of care, which is essential in healthcare settings [2, 3, 30]. Furthermore, working conditions that ensure health and safety help reduce physical and mental stress, decrease the risk of injuries, and promote overall wellbeing, contributing to nurses’ ability to provide high-quality care [1, 3]. Finally, reliable feedback on performance helps nurses improve their skills and recognize their contributions [2, 31].

While each dimension of enriched work design can improve specific aspects of the work experience, the aggregate impact tends to create a more holistic and supportive work environment that optimizes employee wellbeing and performance [11, 12]. The combination of characteristics often leads to synergies that enhance overall outcomes more effectively than any single dimension on its own [32]. Synergistic effects occur when the positive impact of multiple job characteristics interacts to produce greater overall benefits, such as increased job satisfaction, wellbeing, performance, and reduced burnout [6, 31–33]. Several previous studies highlight these synergistic effects. Hackman and Oldham’s model [10] posits that multiple job characteristics, such as skill variety, task identity, task significance, autonomy, and feedback, work together to create meaningful work experiences. They suggest that when these characteristics are all present in a job, they lead to higher motivation, satisfaction, and performance than any individual characteristic would in isolation. Recent research [6, 31, 32] emphasizes that enriched work designs involve the interplay of different factors like autonomy, feedback, and social support. These factors do not just add value individually, but interact to enhance employee engagement, learning, and wellbeing. For instance, autonomy combined with feedback fosters a learning environment, which supports professional development and better problem-solving. Another study [34] found that job resources such as autonomy, support, and opportunities for growth buffer against job demands and prevent burnout. This protective effect is particularly strong when multiple dimensions are present, highlighting the synergistic relationship between them [24]. Research in healthcare settings [35], found that when job autonomy is combined with supportive leadership and meaningful feedback, nurses experience lower levels of burnout and higher job satisfaction. The combination of these factors contributes to a more supportive environment, where nurses can thrive, and patient care quality improves. Overall, the evidence suggests that the synergistic effects of multiple job characteristics in an enriched work design is a critical component of a supportive work environment for wellbeing and optimize performance, which ultimately benefits patient care.

### Psychological Safety in Nurses’ Work Environment

A work environment that promotes psychological safety enables nurses to voice concerns, report errors, and suggest improvements without fear of punishment, fostering an atmosphere of trust and continuous learning [36–39]. Psychological safety is a key positive antecedent of nurses’ performance [40]. Psychological safety refers to the extent to which nurses feel safe to take interpersonal risks, such as speaking up, surfacing concerns, or disagreeing openly, without fear of negative repercussions. It is considered crucial for nurses’ performance due to its impact on individual and team dynamics within healthcare settings [22, 41]. In psychologically safe environments, nurses are more likely to seek feedback, learn from mistakes, and engage in both professional development and team learning. Research also indicates that psychological safety contributes to a positive work environment, promoting nurses’ emotional wellbeing and job satisfaction [22], as well as serving as a buffer against burnout [41]. When nurses feel psychologically safe, they are more likely to communicate effectively with colleagues, leading to better coordination of care. This, in turn, positively influences patient safety and the overall quality of care [36, 37]. Furthermore, psychological safety acts as a mediator between various antecedents, such as leadership styles and work design characteristics, and behavioral outcomes, such as team learning behavior and task performance [15, 38, 39, 42–44]. For example, leadership can encourage psychological safety, creating the conditions for creative problem‐solving and learning from errors [42, 43].

Previous research indicates that work design characteristics play a critical role in fostering psychological safety at work. Job Characteristics Theory proposes that work design has significant impact on employees’ psychological states [10]. Specifically, Edmondson’s seminal work [38] shows that task design, social support, skill variety, and feedback serve as antecedents of psychological safety. A later meta-analysis confirms that work design features like autonomy, job enrichment, and supportive work contexts significantly contribute to psychological safety by signaling trust and collaboration [39].

### The Role of Leadership in Shaping Work Environments

Leadership is a pivotal factor in shaping the work environment and, by extension, the wellbeing and performance of nurses [15–17]. Different leadership styles—such as transformational, inclusive, and abusive leadership—have varying impacts on the work environment. Transformational leadership, characterized by inspiring and motivating employees to exceed their expectations and focusing on individual development, has been shown to positively influence nurse satisfaction, reduce burnout, and enhance patient care quality [16]. Inclusive leadership, which promotes a sense of belonging and values diversity, has similarly been linked to enhanced psychological safety and job satisfaction [15]. On the other hand, leadership behaviors associated with abusive supervision or toxic leadership can have detrimental effects on the work environment, leading to increased stress, reduced job satisfaction, and higher turnover intentions among nurses [17, 45]. Effective leadership is essential for fostering a positive work environment that supports nurse wellbeing, enhances teamwork, and promotes high standards of patient care.

### Leaders’ Dark Triad Traits Impact on Nurses’ Work Design Perceptions and Psychological Safety

A less studied facet of leadership characteristics is the Dark Triad personality traits. Research indicates that the three Dark Triad traits—Machiavellianism, narcissism, and psychopathy—while interrelated, represent conceptually distinct constructs encompassing different aspects of antisocial behavior [46]. Prior literature has identified a “core” component of shared variance among these traits, with proposed commonalities including lack of empathy or callousness, primary psychopathy components, and tendencies toward social dominance and power-seeking behavior [46]. Leaders who exhibit these traits often create toxic work environments characterized by fear, hostility, and mistrust, which can significantly impact employees’ psychological safety, job satisfaction, career fulfillment, and overall wellbeing [17–20]. Within organizational settings, these traits have been consistently associated with increased workplace stress, diminished morale, and elevated turnover rates [47–49].

Recent studies beyond the healthcare sector have demonstrated that managers with pronounced Dark Triad traits negatively influence various employee outcomes, including job satisfaction, turnover intentions, career progression, wellbeing, and team performance. For example, subordinates working under managers with Dark Triad characteristics may engage in knowledge hiding as a defensive response to perceived threats [50]. These managers frequently manipulate task assignments, limit employee autonomy, and withhold positive feedback, resulting in increased stress and demoralization among their staff [17, 51, 52]. Furthermore, Dark Triad traits in supervisors, along with other forms of destructive leadership, typically undermine followers’ perceptions of crucial job characteristics, such as decision-making autonomy, social support, and feedback quality [45, 53, 54], ultimately compromising followers’ career success and wellbeing [55].

In healthcare, where the quality of patient care is paramount, the presence of Dark Triad traits in leadership roles could have particularly damaging effects. Leaders with these traits may undermine collaboration, discourage open communication, and foster a culture of blame, all of which are detrimental to patient safety, quality of care, and the collaborative and continuous learning of nursing work.

### Current Study: Testing a Multiple Mediation Model

Although the negative effects of Dark Triad traits have been well-documented across various sectors [19–21, 26, 27], research examining their specific impact in healthcare settings remains limited. Given the unique demands of healthcare—such as collaboration, compassion, and patient care—the harmful consequences of these traits could be particularly critical. Understanding how managers’ Dark Triad traits influence nurses’ psychological safety and work design is therefore essential for fostering environments that support both performance and wellbeing.

**FIGURE 1:**
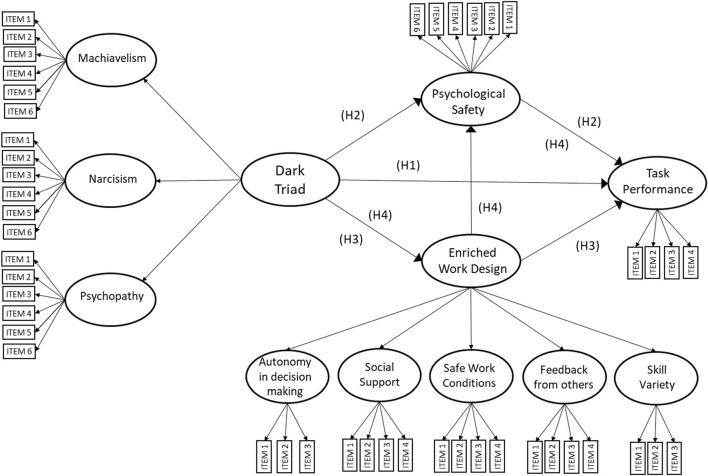
Structural equation model of the direct effects of supervisors’ dark triad traits on nurses’ task performance (H1), and the indirect effects mediated by psychological safety and enriched work design (H2, H3, H4) (Chile, 2017–2019).

This study explores how supervisors’ Dark Triad traits interact with work design to affect nurses’ psychological safety and task performance. Figure 1 presents a structural equation model that incorporates two key latent variables: the core factor of supervisors’ Dark Triad traits and the second-order factor of enriched work design characteristics. By modeling DT traits as a second-order factor, this approach captures the collective toxic influence of Machiavellianism, narcissism, and psychopathy on work environment perceptions and performance outcomes [46]. Similarly, treating work design as a second-order construct reflects the combined effects of autonomy, skill variety, social support, and feedback in shaping nurses’ experiences and performance [24, 32, 34].

Based on previous literature discussed above, three mediation pathways are proposed in the structural equation model in Figure 1 to explain how supervisors’ Dark Triad traits, work design, and psychological safety interact to influence task performance:

First, because supervisors’ Dark Triad personality traits can negatively influence nurses’ task performance, and because other mediation mechanisms not fully accounted for by the model have documented (e.g., increased stress, burnout, or reduced morale), it is proposed that some of the impact of supervisors’ Dark Triad traits will have a direct effect on nurses’ task performance (H1).

H1 Supervisors’ Dark Triad personality traits are negatively related to nurses’ task performance.

The first mediation hypothesis managers’ Dark Triad traits are expected to negatively affect nurses’ psychological safety. Leaders’ high in Dark Triad traits may foster an environment where nurses feel unsafe to express concerns or take risks, fearing negative repercussions. This diminished psychological safety reflects a work environment where interpersonal risks are perceived to carry negative consequences, eroding trust and open communication. In such settings, nurses are less likely to perform effectively, as they lack the confidence and psychological security needed for optimal task engagement. Consequently,

H2 Psychological safety mediates the relationship between managers’ Dark Triad traits and nurses’ performance.

The second mediation path focuses on the impact of managers’ Dark Triad traits on nurses’ perceptions of their work design. Supervisors exhibiting such toxic traits are anticipated to negatively affect key dimensions of nurses’ work design, such as autonomy in decision-making, social support, feedback from others, skill variety, and safe work conditions. These dimensions are essential for fostering an enriched work environment. However, when supervisors limit autonomy, provide insufficient feedback, or fail to support a safe work setting, nurses’ perceptions of their work design deteriorate. This compromised work design is expected to result in lower task performance, as an enriched work environment is known to enhance engagement, job satisfaction, and overall effectiveness. Therefore,

H3 Enriched work design mediates the relationship between managers’ Dark Triad traits and nurses’ performance.

The third mediation hypothesis posits that nurses’ perceptions of an enriched work design positively influence their psychological safety, which in turn improves task performance. When nurses perceive high levels of autonomy, skill variety, social support, safe working conditions, and meaningful feedback, these elements synergistically enhance their psychological safety, facilitating an environment of empowerment, trust, and respect. For example, autonomy enables nurses to feel in control of their work, while skill variety boosts their confidence in their competencies. Strong social support fosters trust and collaboration, making nurses feel valued and respected. Safe working conditions allow them to focus on their tasks without distractions or fears regarding their wellbeing. Meaningful feedback reinforces that their contributions are appreciated, encouraging open communication. Collectively, these factors create a supportive atmosphere where nurses feel secure to express concerns and take interpersonal risks, ultimately leading to improved task performance.

H4 Managers’ Dark Triad traits negatively influence work design, which in turn affects psychological safety and ultimately impacts performance.

The following sections will present the methodology used to test these hypotheses, along with the results and discussion.

## Methods

### Procedure

To test the proposed model a multisource approach was employed, where nurses work design and psychological safety was reported by nurses, while nurses’ managers Dark Triad traits and nurses’ task performance were reported by nurses’ direct managers. The usage of different sources of information when collecting data from study measures decreases potential common method variance [56]. Each participant provided a written informed consent, acknowledging the voluntary nature of participation, confidentiality of information, and understanding the benefits and risks associated with collaboration.

### Sample

A convenience sampling method was utilized across various healthcare centers and settings where nursing professionals were employed. The healthcare institutions size was classified by managers as belonging to the following ranges of number of employees (0–100: 38%, 101–500: 21%, 501–1,000: 18%, 1,001–5,000: 15%, 5,001–10,000: 3%, >10,000: 5%). Managers also classified if their healthcare organizations were part of the public sector (45.3%) or private sector (54.7%), and if their organizations were a profit (31.6%) or non-profit (68.4%) organization. A total of 256 manager-nurse pairs responded with a complete data set and qualified for the study requirements. Inclusion criteria for nurses involved being a nursing professional without a managerial role, expressing interest in participation, and consenting to the informed consent. For nurse managers, inclusion criteria involved being the direct supervisor of participating nursing professionals for at least 6 months, demonstrating interest in participation, and accepting to the informed consent. Managers average age was of 51.51 years old, being 83.6% females, and team members averaged 39.65 years old in age, 87.9% of them being female. Average tenure of managers at their job position was 6.24 years, while average tenure of nurse workers was 4.18 years.

### Measures

Five key dimensions of nurses’ work design, including autonomy in decision making, social support, feedback from others, skill variety, and safe work conditions were measured using a Spanish adaptation [57] of The Work Design Questionnaire (WDQ) [58]. Dark Triad traits were measured in supervisors using six items for each triad dimension (Machiavellianism, narcissism, and psychopathy) using a self-assessment version of D3-Short questionnaire [59]. Psychological Safety was reported by nurses through Edmondson’s scale [38], evaluating the extent to which nurses feel safe to take interpersonal risks in their work without fear of negative consequences. A four-item scale measuring nurses task performance was adapted from previous research [60, 61]. This supervisor-rated scale focused on nurses’ effectiveness, excellence, quality of care, and adequacy in fulfilling their job responsibilities. All measures used a Likert-type response format with five rating points, where 1 indicates total disagreement and 5 indicates total agreement. We controlled nurses’ education level, job and organization tenure, and age, which has been previously related to nurses’ performance [1]. As well, we controlled the private/public, profit/non-profit nature of organizations, and organizational size, measured by number of employees.

Item translation for all surveys (except WDQ) was conducted using a separate translator, reviewer, and receptor [62] – all of whom were proficient in both English and Spanish. We conducted a pilot study with a small sample of respondents who judged the readability and comprehension of the translated version. Pilot participants’ concerns were then discussed by the researchers, and appropriate changes were made to the final version of the surveys.

## Results

Statistical Analyses were made using SPSS 28 software for descriptive statistics, AMOS 28 software to perform confirmatory factor analyses, and to test the structural equation model (SEM) in Figure 1. As seen in Table 1, Cronbach’s alpha reliability for all scales is above the 0.70 cut-off score, and therefore shows internal consistency [63]. The values for skewness (max = 0.10; min = −1.03) and kurtosis (max = 1.54; min = −1.07) for all items range between −2 and +2 – values, which are considered acceptable to indicate normal univariate distributions [64]. Correlation analyses show a significant positive relationship of all work design dimensions with psychological safety and with nurses’ task performance. Table 1 also shows a negative relation of the three components of Dark Triad with psychological safety. A negative relation is also significant between managers psychopathy trait and task performance, while Machiavellianism and narcissism traits had a negative but non-significant relation with nurse performance. The correlations of managers Dark Triad traits with all work design dimensions were in the expected direction, all negative, but not all of them achieved statistical significance.

**TABLE 1:** Means, standard deviations and correlations for all variables (Chile, 2017–2019).

	*M*	*SD*	1	2	3	4	5	6	7	8	9	10	11
1. Autonomy in decision making	3.71	0.70	(0.76)										
2. Social Support	4.04	0.72	0.27	(0.77)									
3. Skill variety	3.65	0.73	0.34	0.29	(0.85)								
4. Feedback from others	3.43	0.81	0.34	0.33	0.24	(0.78)							
5. Safe work conditions	3.96	0.73	0.24	0.35	0.21	0.27	(0.73)						
6. Machiavellianism	2.11	0.73	−0.17	−0.02	−0.09	−0.13	−0.08	(0.87)					
7. Narcissism	2.58	0.74	−0.15	−0.09	−0.09	−0.12	−0.13	0.50	(0.84)		—		
8. Psychopathy	1.63	0.57	−0.13	−0.12	−0.14	−0.11	−0.08	0.66	0.48	(0.81)			
9. Psychological Safety	3.69	0.73	0.18	0.31	0.23	0.31	0.24	−0.29	−0.19	−0.28	(0.88)		
10. Task Performance	3.98	0.70	0.12	0.31	0.24	0.20	0.18	−0.09	−0.04	−0.26	0.36	(0.87)	
11. Public (1)/Private (2) sector	1.55	0.50	−0.15	−0.03	−0.13	−0.14	−0.07	0.12	0.08	0.06	−0.11	−0.01	
12. Profit (1)/Nonprofit (2)	1.68	0.47	−0.04	0.06	−0.02	0.09	0.08	−0.00	0.07	0.07	0.04	0.05	−0.16

Note: Correlations > |0.12| are significant at *p* < 0.05; correlations > |0.16| are significant at *p* < 0.01.

**FIGURE 2:**
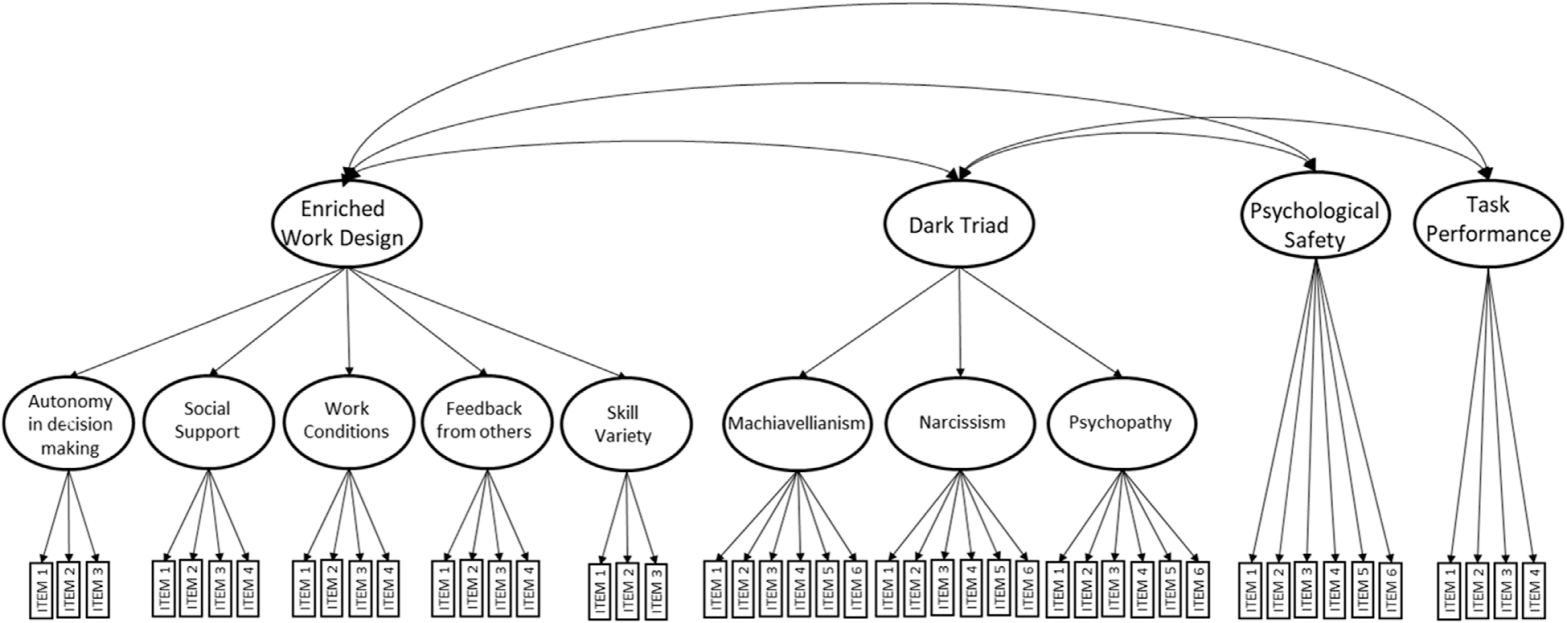
The best-fitting Confirmatory Factor Analysis model CFA-3 (Chile, 2017–2019). Note: Model CFA-3 organizes work environment items into five first-order dimensions (autonomy in decision-making, social support, skill variety, safe work conditions, and feedback from others), which collectively contribute to an overall second-order work environment factor. Dark Triad items are grouped into three first-order factors (Machiavellianism, narcissism, and psychopathy), contributing to an overall second-order Dark Triad factor. Psychological safety and task performance items are assigned to two additional first-order factors. (CMIN/DF = 1.467; IFI = 0.910; CFI = 0.909; RMSEA = 0.043; LO90: 0.038, HI90: 0.047).

A two-stage analysis was performed on data. First, a CFA was performed to test the construct validity of the measures, followed by structural equation modeling (SEM) analyses intended to test structural models [65]. Three CFA models were tested, and results are presented in Table 2. Model CFA-1 tested all items in the study in a single factor model achieving a poor fit (CMIN/DF = 4.28, CFI = 0.36, IFI = 0.35, RMSEA = 0.11). Model CFA-2 tested all items grouped into the four main factors of the study (work design, psychological safety, Dark Triad and work performance) which also shows a poor fit (CMIN/DF = 2.55, CFI = 0.70, IFI = 0.69, RMSEA = 0.08). Model CFA-3 departs from model CFA-2 in that it arranges work design items into five first order dimensions (autonomy in decision making, social support, safe work conditions, skill variety and feedback from others) feeding a second order enrichened work design latent variable. Similarly, Dark Triad items are arranged into three first order factors (Machiavellianism, Narcissism and Psychopathy) which feed an overall second order Dark Triad latent variable. As seen in Figure 2, model CFA-3 has a much better fit that the other two CFA models (CMIN/DF = 1.467, CFI = 0.910, IFI = 0.909, RMSEA = 0.043), thus giving support to the Dark Triad and enriched work design latent variables.

**TABLE 2:** Confirmatory factor analysis results comparing models with one factor, four factors, and a combination of second-order and first-order factors (Chile, 2017–2019).

	χ^2^	df	CMIN/DF	CFI	IFI	RMSEA	LO 90/HI 90
Model 1	4229.54	989	4.277	0.355	0.349	0.113	0.110/0.117
Model 2	2510.40	893	2.554	0.696	0.693	0.078	0.074/0.082
Model 3	1428.60	954	1.467	0.910	0.909	0.043	0.038/0.047

Note: Model 1 is a single-factor model. Model 2 includes four distinct factors: enriched work design, Dark Triad traits, psychological safety, and task performance. In Model 3, the enriched work design encompasses five first-order factors: autonomy, social support, skill variety, safe work conditions, and feedback. The Dark Triad traits comprise three first-order factors: Machiavellianism, narcissism, and psychopathy, along with additional first-order factors for psychological safety and task performance. All χ^2^ values significant at *p* < 0.01.

**FIGURE 3:**
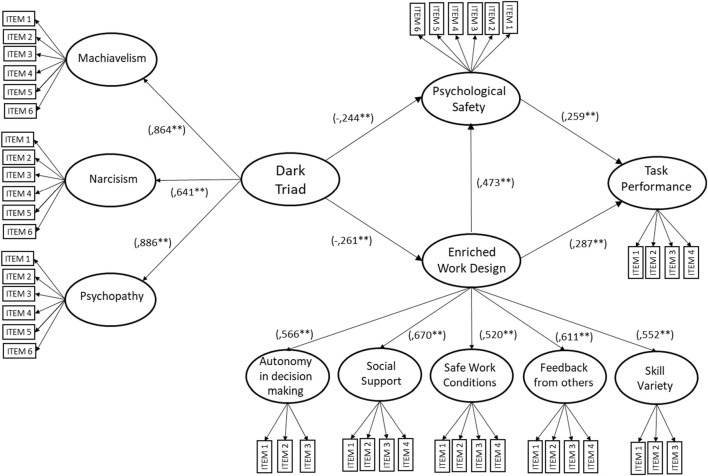
Standardized coefficients for Structural Equation Model 2 predicting the indirect effects of Supervisors’ Dark Triad Traits on Nurses’ Task Performance, mediated by Psychological Safety and Enriched Work Design (CMIN/DF = 1.494; IFI = 0.904; CFI = 0.903; RMSEA = 0.044; LO90: 0.039, HI90: 0.049). Standardized Regression Weights in parentheses, **p < 0.01 (Chile, 2017–2019).

Model SEM-1 tests the mediation hypotheses and direct effects proposed in this study (see Figure 1). Model SEM-1 shows a good fit of the data (CMIN/DF = 1.494; IFI = 0.904; CFI = 0.903; RMSEA = 0.044; HI90: 0.039 HI90: 0.049). Table 3 presents the effect size and significance for the direct and indirect paths in the model. All model paths are significant except for the direct effect of Dark Triad traits on task performance, which provides no support for H1. The indirect effect of Dark Triad traits on task performance through the psychological safety mediator is small but significant (effect size: −0.053; 95% Bootstrap Confidence Interval: [−0.114, −0.002]), providing support for H2. Additionally, the indirect effect of Dark Triad traits on task performance through the enriched work design mediator (H3) is also significant (effect size: −0.0599; 95% Bootstrap Confidence Interval: [−0.1151, −0.0142]). Finally, the serial mediation path, from Dark Triad traits through enriched work design, followed by psychological safety and leading to task performance, is significant as well (effect size: −0.053; 95% Bootstrap Confidence Interval: [−0.114, −0.002]), supporting H4. A model SEM-2 was tested, identical to model SEM-1, except that the direct path of Dark Triad on performance was eliminated, while retaining all other direct and mediation hypotheses of this study. No changes occurred to the model fit indexes (CMIN/DF = 1.493; IFI = 0.904; CFI = 0.903; RMSEA = 0.044), despite slightly strengthen significance for the rest of the model paths (see Figure 3).

**TABLE 3:** Analysis of direct effects and multiple mediation paths proposed in model 1: estimates and significance (Chile, 2017–2019).

Path	Standardized estimate (β)	Effect size	Standard error	*p*-value	Bootstrap confidence intervals (95%)	Significance
Direct Effects						
H1: DT → TP	−0.065	−0.089	0.102	0.384	[−0.289, 0.134]	Not Significant
Indirect Effects						
H2: DT → PS → TP	−0.058	−0.079	0.041	0.006	[−0.188, −0.019]	Significant
H3: DT → EWD → TP	−0.071	−0.097	0.063	0.013	[−0.287, −0.018]	Significant
H4: DT → EWD → PS → TP	−0.028	−0.039	0.023	0.007	[−0.111, −0.009]	Significant
Total Indirect Effects	−0.158	−0.216	0.075	0.001	[−0.423, −0.105]	Significant
Total Effects	−0.223	−0.304	0.184	0.025	[−0.777, −0.035]	Significant

Note: DT, dark triad traits; TP, task performance; PS, psychological safety; EWD, enriched work design.

Because correlations of Dark Triad dimensions were not all significant with task performance (see Table 1), we adapted model SEM-1 to separately test for the Machiavellianism, narcissism, and psychopathy traits, in replacement for the overall Dark Triad second order dimension. Post-hoc results indicate that when testing for the Machiavellianism trait alone, the mediation model has a good fit (CMIN/DF = 1.51, IFI = 0.93, CFI = 0.93 RMSEA = 0.045). However, the direct effect of Machiavellianism trait on task performance (β = −.23, *p* > 0.712). Similarly, when testing for the narcissism trait alone, the mediation model also has a good fit (CMIN/DF = 1.35, IFI = 0.99, CFI = 0.99 RMSEA = 0.043), but the direct effect of narcissism trait on task performance is not significant (β = 0.08, *p* > 0.156). In the case of the psychopathy trait, our data supported the mediation model (CMIN/DF = 1.53, IFI = 0.92, CFI = 0.92 RMSEA = 0.046). Interestingly, the direct effect of psychopathy traits on task performance is significant (β = −.27, *p* < 0.001).

Furthermore, since not all correlations between work design dimensions and Dark Triad traits were significant (see Table 1), we adapted SEM Model 1 *post hoc*, testing each work design dimension (autonomy, social support, feedback, skill variety, and safe work conditions) independently as mediators. Results for these direct and indirect paths indicate that none of the direct paths from Dark Triad traits to task performance were significant: autonomy (β = −0.102, 95% CI [−0.589, 0.169], *p* = 0.327), social support (β = −0.128, 95% CI [−0.521, 0.114], *p* = 0.212), skill variety (β = −0.074, 95% CI [−0.051, 0.157], *p* = 0.473), safe work conditions (β = −0.076, 95% CI [−0.305, 0.126], *p* = 0.485), and feedback (β = −0.083, 95% CI [−0.319, 0.120], *p* = 0.428). Thus, Dark Triad traits did not directly influence nurses’ task performance.

However, the indirect paths from Dark Triad traits to task performance via psychological safety were significant for all dimensions: autonomy (β = −0.173, 95% CI [−0.324, −0.086], *p* < 0.001), social support (β = −0.133, 95% CI [−0.263, −0.057], *p* < 0.001), skill variety (β = −0.147, 95% CI [−0.282, −0.073], *p* < 0.001), safe work conditions (β = −0.165, 95% CI [−0.312, −0.081], *p* < 0.001), and feedback (β = −0.147, 95% CI [−0.289, −0.068], *p* < 0.001). These results suggest psychological safety mitigates the negative effects of Dark Triad traits on task performance.

Finally, the direct paths from Dark Triad traits to task performance via specific job dimensions were not significant: autonomy (β = −0.008, 95% CI [−0.065, 0.029], *p* = 0.536), social support (β = −0.03, 95% CI [−0.131, 0.018], *p* = 0.184), skill variety (β = −0.04, 95% CI [−0.145, 0.000], *p* = 0.047), safe work conditions (β = −0.02, 95% CI [−0.119, 0.014], *p* = 0.22), and feedback (β = −0.019, 95% CI [−0.085, 0.005], *p* = 0.132). However, in the original model (Figure 1), where all work design dimensions were combined into an enriched work design second-order factor, the indirect mediation was significant (see Table 3), indicating that the combined effects of work design components are more influential than individual dimensions.

## Discussion

This study aimed to elucidate the mechanisms by which managers’ Dark Triad traits (Machiavellianism, narcissism, and psychopathy) affect nurses’ psychological safety, perceptions of enriched work design, and task performance in healthcare settings. The findings revealed significant mediation effects of psychological safety and work design on the relationship between Dark Triad traits and task performance, suggesting that these traits adversely influence performance primarily through their impact on work environment factors.

### Theoretical Implications

The results underscore the critical role of psychological safety as a mediator in the relationship between managers’ Dark Triad traits and nurses’ task performance. Managers with elevated Dark Triad traits tend to create environments where nurses feel psychologically unsafe, inhibiting optimal performance. This finding aligns with Edmondson’s theory, which posits that psychological safety is crucial for fostering high performance [38]. Our study demonstrates that the Dark Triad traits disrupt psychological safety, emphasizing the importance of maintaining a supportive work environment to enhance performance outcomes.

The identification of enriched work design as another significant mediator reinforces the idea that toxic leadership degrades essential job characteristics. Enriched work design is pivotal not only for psychological safety but also for task performance, as supported by job characteristics theory [10]. The synergistic effects of enriched work design, rather than isolated job dimensions, appear to buffer the adverse impact of toxic leadership. This holistic view highlights the importance of integrating job design improvements with leadership development initiatives.

Interestingly, despite the presence of managers with Dark Triad traits, nurses’ task performance was still predicted in a positive direction. This suggests that while toxic traits negatively affect work conditions, the mediating effects of psychological safety and enriched work design can help buffer these influences. Research supports the idea that supportive environments foster resilience, enabling employees to perform effectively even under adverse leadership conditions [12, 15, 38]. These mediators act as protective factors, ensuring that positive work environment elements override negative leadership traits, leading to improved task performance.

*Post-hoc* analyses revealed that psychopathy had a direct, significant effect on performance, distinguishing it from Machiavellianism and narcissism. While the latter traits primarily exert their influence through mediation pathways, psychopathy seems to have a more immediate and detrimental impact. This finding supports prior research identifying psychopathy as the most toxic of the Dark Triad traits [21, 46]. Additionally, the confirmation of a second-order factor for enriched work design, and the significance of its mediation, suggests that these job characteristics function as an integrated construct, collectively influencing outcomes [24, 32, 34, 35].

Lastly, by extending the examination of Dark Triad traits to the healthcare sector, this study contributes to literature that has primarily focused on other industries. The absence of direct effects from Dark Triad traits—except for psychopathy—challenges prior assumptions of their uniformly negative impact [21], prompting a more nuanced understanding of their relationship with task performance.

### Practical Implications

The findings hold significant implications for healthcare management. To mitigate the adverse effects of toxic leadership, organizations should prioritize initiatives aimed at promoting psychological safety and enriched work environments. Leadership development programs must focus on identifying and addressing Dark Triad traits among managers. Additionally, fostering environments where nurses experience an enriched work design is essential for enhancing performance and overall job satisfaction. The confirmation of second-order factors for Dark Triad and enriched work design characteristics suggests that interventions aimed at mitigating the effects of the Dark Triad or enhancing work design may benefit from a holistic approach that addresses these constructs as integrated entities rather than isolated traits or dimensions.

### Strengths, Limitations and Future Research

This study’s strength lies in its multisource data collection approach, which minimizes biases associated with common method variance, and the diverse sample drawn from various healthcare centers, enhancing the generalizability of the findings. However, limitations include the reliance on convenience sampling, which may restrict the representativeness of the results, and the focus on specific mediating factors without exploring others, such as emotional exhaustion or stress. Future research could incorporate these variables to further elucidate the direct effects of toxic leadership traits.

Future research should explore other mechanisms by which Dark Triad traits affect employee performance, including emotional exhaustion and burnout. Investigating the role of individual nurse characteristics—such as resilience or coping strategies—as moderators could provide valuable insights into the interplay between managerial traits and employee outcomes. Given the significant negative impact of psychopathy on task performance observed in this study, further research is warranted to understand how this trait uniquely affects healthcare settings compared to Machiavellianism and narcissism.

### Conclusion

In conclusion, this study illuminates the pathways through which managers’ Dark Triad traits impact nurses’ psychological safety, work design, and performance. The findings emphasize the urgent need for healthcare organizations to address toxic leadership behaviors and cultivate supportive, well-designed work environments that promote high performance among nurses. Such efforts will not only enhance employee wellbeing but also improve patient care outcomes.
